# TRAM deletion attenuates monocyte exhaustion and alleviates sepsis severity

**DOI:** 10.3389/fimmu.2023.1297329

**Published:** 2023-12-15

**Authors:** Jing Wang, Yajun Wu, RuiCi Lin, Yao Zhang, Liwu Li

**Affiliations:** Department of Biological Sciences, Virginia Tech, Blacksburg, VA, United States

**Keywords:** monocyte exhaustion, TRAM, sepsis, intervention, signaling

## Abstract

Monocyte exhaustion characterized by immune-suppressive features can develop during sepsis and contribute to adverse patient outcomes. However, molecular mechanisms responsible for the establishment of immune-suppressive monocytes with reduced expression of immune-enhancing mediators such as CD86 during sepsis are not well understood. In this study, we identified that the TLR4 intracellular adaptor TRAM plays a key role in mediating the sustained reduction of CD86 expression on exhausted monocytes and generating an immune-suppressive monocyte state. TRAM contributes to the prolonged suppression of CD86 through inducing TAX1BP1 as well as SARM1, collectively inhibiting Akt and NFκB. TRAM deficient mice are protected from cecal slurry-induced experimental sepsis and retain immune-competent monocytes with CD86 expression. Our data reveal a key molecular circuitry responsible for monocyte exhaustion and provide a viable target for rejuvenating functional monocytes and treating sepsis.

## Introduction

Sepsis caused by polymicrobial infections poses a major health concern for patients in critical care units around the world, with high risks of morbidity and mortality (1). Dysfunctions of exhausted monocytes in both experimental sepsis and human septic patients drastically compromise the host capability in effectively containing tissue inflammation and anti-microbial defense (2–4). Exhausted monocytes exhibit sustained expression of pathogenic inflammatory mediators, and down-regulate immune-enhancing mediators, collectively leading to pathogenic inflammation and immune suppression (4, 5). Despite its fundamental and clinical relevance, underlying molecular mechanisms are not thoroughly understood.

Key microbial associated danger-pattern molecules such as lipopolysaccharide (LPS) are known to trigger robust innate immune responses via Toll-like-receptor 4 (TLR4) signaling processes (6, 7). Independent studies reveal that the TRAM adaptor is vitally responsible for the initial cellular response to LPS challenge (4, 8, 9). TRAM-mediated activation of transcription factors such as NFκB and STAT1 are responsible for the expression of inflammatory mediators (10, 11). Following the initial response, prolonged exposure of monocytes to septic high dose of LPS challenges exhibit an altered landscape of gene expression, with preferential expression of selected pathogenic genes such as iNOS, S100A8 and CD38 as well as suppression of immune-enhancing genes such as CD86 (4, 5, 12). The sustained activation of STAT1 is responsible for the persistent activation of pathogenic inflammatory mediators (4, 13). On the other hand, a reduction of NFκB and Akt signaling is responsible for reduced expression of immune-enhancing genes such as CD86. Although TRAM is shown to contribute to the sustained activation of STAT1 in monocytes exposed to prolonged LPS challenge, molecular mechanisms responsible for the suppression of NFκB/Akt and CD86 expression are not well understood.

We recently reported that monocyte exhaustion can be recapitulated *in vitro*, by culturing monocytes with a drastic and prolonged challenge of high dose bacterial endotoxin lipopolysaccharide (LPS) (4). *In vitro* exhausted monocytes exhibit key characteristics of pathogenic inflammation and immune suppression, as compared to septic monocytes collected from both experimental animals and human patients (4). Using this well-defined *in vitro* culture model, we herein examined the role of TRAM and underlying mechanisms during the suppression of Akt/NFκB and CD86 expression in exhausted monocytes. Complementing our *in vitro* mechanistic studies, we further examined the role of TRAM in suppressing CD86 expression *in vivo*, by employing the cecal slurry-mediated sepsis model.

## Results

### The generation of sustained monocyte exhaustion requires TRAM adaptor signaling

**Figure 1:**
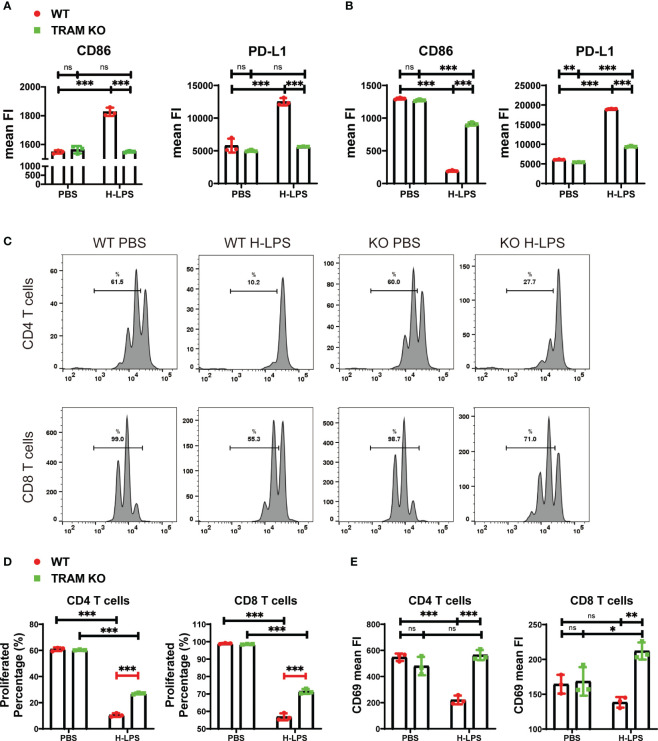
The generation of sustained monocyte exhaustion requires TRAM adaptor signaling. **(A, B)** BMDMs from WT and TRAM KO mice were stimulated with either PBS or H-LPS (100 ng/mL) for 5 hours or 5 days. The mean FIs of CD86 and PD-L1 in WT and TRAM KO monocytes on 5 hours **(A)** or 5 days **(B)** were analyzed with flow cytometry. The relative changes in mean FIs of CD86 and PD-L1 comparing LPS *vs* PBS-treated monocytes were determined and plotted in the graphs. **(C–E)** Splenic T cells were cocultured with 5-day PBS or H-LPS treated WT or TRAM KO monocytes for 2 days. CFSE prelabeled recipient T cells were stained for CD4, CD8, and CD69 and analyzed by flow cytometry at day 2. The representative CFSE histogram of CD4^+^ and CD8^+^ T cells were shown in **(C)**. The quantification of CD4^+^ and CD8^+^ T cells proliferation percentage were shown in **(D)**. Both CD4^+^ and CD8^+^ T cells were divided more than 3 times after coculture with PBS treated WT or TRAM KO monocytes. **(E)** The CD69 expressions of CD4^+^ and CD8^+^ T cells after 2-day coculture were analyzed with flow cytometry. The data are representative of at least three independent experiments, and error bars represent means ± SD. ns, not significant, *p<0.05, **p<0.01, ***p<0.001.

As we reported (4), monocytes following prolonged LPS challenges develop a signature state of exhaustion, with reduced expression of immune-enhancing genes such as CD86 while maintaining the expression of immune-suppressive genes such as PD-L1. We not only validated this phenotype in this current study (**Figure 1**), but also demonstrated that prolonged challenges with LPS are necessary in order to establish monocyte exhaustion. This exhaustion phenotype is distinct from the traditional concept of single dose LPS training. The single dose innate training only involves a transient challenge which induces an initial phase of inflammatory response and a second phase of tolerance (14). Following prolonged LPS challenge, monocytes enter the third and final phase of exhaustion (4) (**Supplementary Figure S1**). In addition to the dampened expression of inflammatory cytokines well-examined in the tolerance scenario (14, 15), exhausted monocytes selectively maintain the robust capability of expressing pathogenic inflammatory genes and immune suppressive genes such as PD-L1, while drastically suppress the expression of immune-enhancing genes such as CD86 (4). As shown in **Figure 1**, naïve monocytes with a short-term LPS treatment (5 h) robustly express both immune-enhancing CD86 as well as immune suppressive PD-L1 (**Figure 1A**). When monocytes were subjected to prolonged LPS challenges for 5 days, the expression levels of immune-enhancing CD86 gene were significantly suppressed, while the immune-suppressive PD-L1 gene were further elevated (**Figure 1B**). Based on our previous report that TRAM is critically involved in LPS sensing (4, 8), we compared the short-term and long-term cellular responses of wild-type and TRAM knockout monocytes to LPS. We confirmed that TRAM is responsible for both the short-term induction of CD86 and PD-L1, but also for the long-term suppression of CD86 in exhausted monocytes.

We next examined the functional consequence of monocyte exhaustion. Consistent with clinical reports that demonstrate immune-suppressive functions of septic monocytes, we observed that *in vitro* exhausted monocytes following prolonged LPS challenge potently suppressed T cell proliferation as well as activation, measured by co-culture assays (**Figures 1C–E**). We further observed that TRAM deficient monocytes exhibit limited suppression of T cells (**Figures 1C–E**).

### TRAM is involved for the sustained suppression of Akt and NFκB in exhausted monocytes leading to reduced expression of CD86

**Figure 2:**
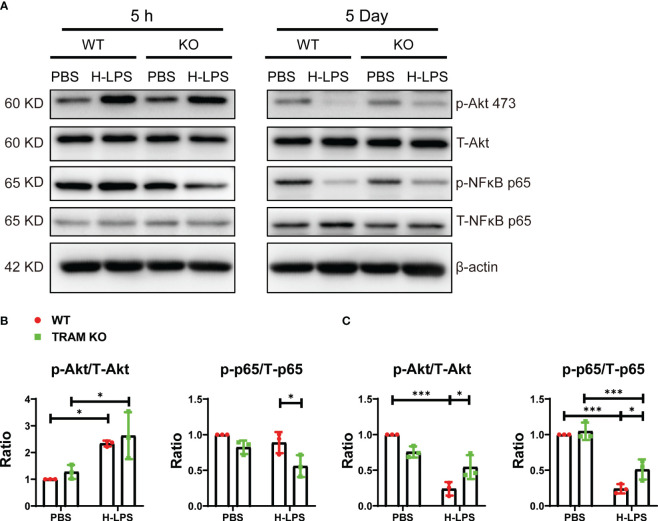
TRAM is involved for the sustained suppression of Akt and NFκB p65 in exhausted monocytes leading to reduced expression of CD86. BMDMs from WT and TRAM KO mice were stimulated with either PBS or H-LPS (100 ng/mL) for 5 hours or 5 days. **(A)** The levels of phosphorylated Akt, total Akt, phosphorylated NFκB p65 and total NFκB p65 were determined by western blot. **(B, C)** The relative levels of p-Akt **(B)** and p-NFκB p65 **(C)** were normalized to T-Akt and T-NFκB p65 separately. Error bars represent means ± SD. *p<0.05, ***p<0.001.

Independent studies in the context of endotoxin tolerance, reveal a reduction of Akt and NFκB p65 in endotoxin tolerance cells, potentially explaining the reduced expression of inflammatory as well as immune-enhancing mediators (16–18). We therefore tested their activation status monocytes treated with short term LPS vs long term LPS. We found that Akt was robustly activated by LPS at the early 5 h stimulation point, correlated with the early induction of CD86 by LPS (**Figures 1A**, **2A, B**). In contrast, as monocytes adapt to the sustained exhaustion state following prolonged LPS treatment, both Akt and NFκB were drastically inhibited, corresponding to suppressed expression of immune-enhancing CD86 (**Figure 1B**). We observed that TRAM deletion attenuated the suppression of Akt as well as NFκB in exhausted monocytes (**Figures 2A, C**). Our data suggest that TRAM is responsible for the sustained suppression of Akt/NFκB and the generation of immune-suppressive exhausted monocytes.

### TRAM is required for the induction of TAX1BP1, a potent suppressor of Akt and NFκB

**Figure 3:**
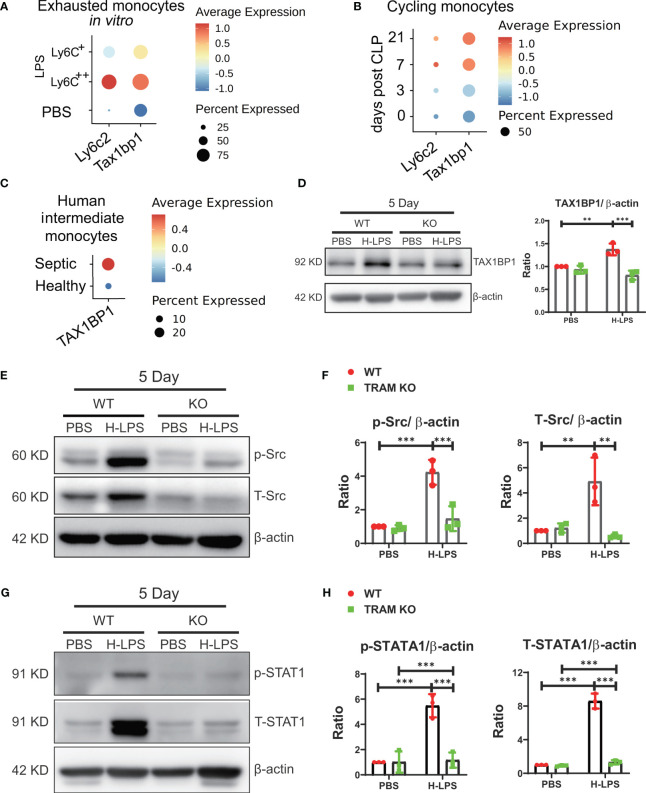
TRAM is required for the induction of TAX1BP1, a potent suppressor of Akt and NFκB. **(A)** BMDMs were treated with either PBS or H-LPS (100 ng/mL) for 5 days and subjected to scRNA-seq analysis using Chromium Single Cell 3’ Reagent Kit V3. The *Ly6c2* and *Tax1bp1* genes levels in PBS control treated (Ly6C^-^) cluster and H-LPS treated clusters (Ly6C^+^, Ly6C^++^) are shown using dot plot. **(B)** Dot plot analysis of day 0, 3, 7, and 21 *Ly6c2* and *Tax1bp1* expression in cycling monocytes from mice after 0, 3, 7, and 21 days post-treatment. **(C)** Dot plot analysis of *Tax1bp1* expression in human intermediate monocytes from healthy control or septic patients. **(D)** BMDMs from WT and TRAM KO mice were stimulated with either PBS or H-LPS (100 ng/mL) for 5 days. The expression level of TAX1BP1 was determined by western blot (left) and the relative levels of TAX1BP1 was normalized to β-actin (right). **(E–H)** BMDMs from WT and TRAM KO mice were stimulated with either PBS or H-LPS (100 ng/mL) for 5 days. The levels of phosphorylated Src, total Src, phosphorylated STAT1 and total STAT1 were determined by western blot **(E, G)**. The relative levels of p-Src, T-Src, p-STAT1 and T-STAT1 were normalized to β-actin separately **(F, H)**. n=3. Error bars represent means ± SD. **p<0.01, ***p<0.001.

We further explored potential mechanisms for the sustained suppression of Akt and NFκB in exhausted monocytes. Through re-analyses of differentially expressed genes of our previous scRNAseq data comparing naïve and exhausted monocytes, we identified TAX1BP1, a previously characterized suppressor of Akt and NFκB (19–21), to be highly induced in exhausted monocytes (**Figure 3A**). We then performed comparative analyses of independently published scRNAseq datasets comparing infiltrating heart monocytes following the onset of cecal ligation and puncture-induced sepsis in experimental animals (22), and observed sustained elevation of TAX1BP1 expression in infiltrating monocytes from septic mice as compared to control mice (**Figure 3B**). Furthermore, it was known that the intermediate CD14CD16 human monocyte subset undergoes expansion in human sepsis patients (23, 24). We then analyzed the TAXBP1 expression levels in the independently published scRNAseq data from healthy and septic peripheral blood monocytes (25), and identified similarly elevated TAX1BP1 expression in the intermediate monocyte subset from septic patients as compared to healthy donors (**Figure 3C**). Based on these comparative analyses, we then performed Western analyses comparing the protein levels of TAX1BP1 in naïve and exhausted murine monocytes. As shown in **Figure 3D**, we indeed observed significantly elevated protein levels of TAX1BP1 in exhausted monocytes. In contrast, the induction of TAX1BP1 was significantly attenuated in TRAM deficient monocytes (**Figure 3D**). TAX1BP1 promoter contains putative STAT1 sites based on search of genebank database. Despite the suppression of Akt and NFκB, we reported that exhausted monocytes exhibit sustained activation of Src and STAT1, responsible for the induction of pathogenic inflammatory mediators (4). We confirmed this finding and observed sustained activation of Src and STAT1 in wild type, but not TRAM deficient monocytes following long-term exhaustion (**Figures 3E–H**). Our data suggest that TRAM-mediated activation of Src/STAT1 enables sustained expression of TAX1BP1, leading to the suppression of Akt/NFκB and the reduced expression of immune-enhancing genes such as CD86 (**Figures 1B**, **2A–C**; **Supplementary Figure S2**).

### TRAM works synergistically with SARM1 in driving monocyte exhaustion

**Figure 4:**
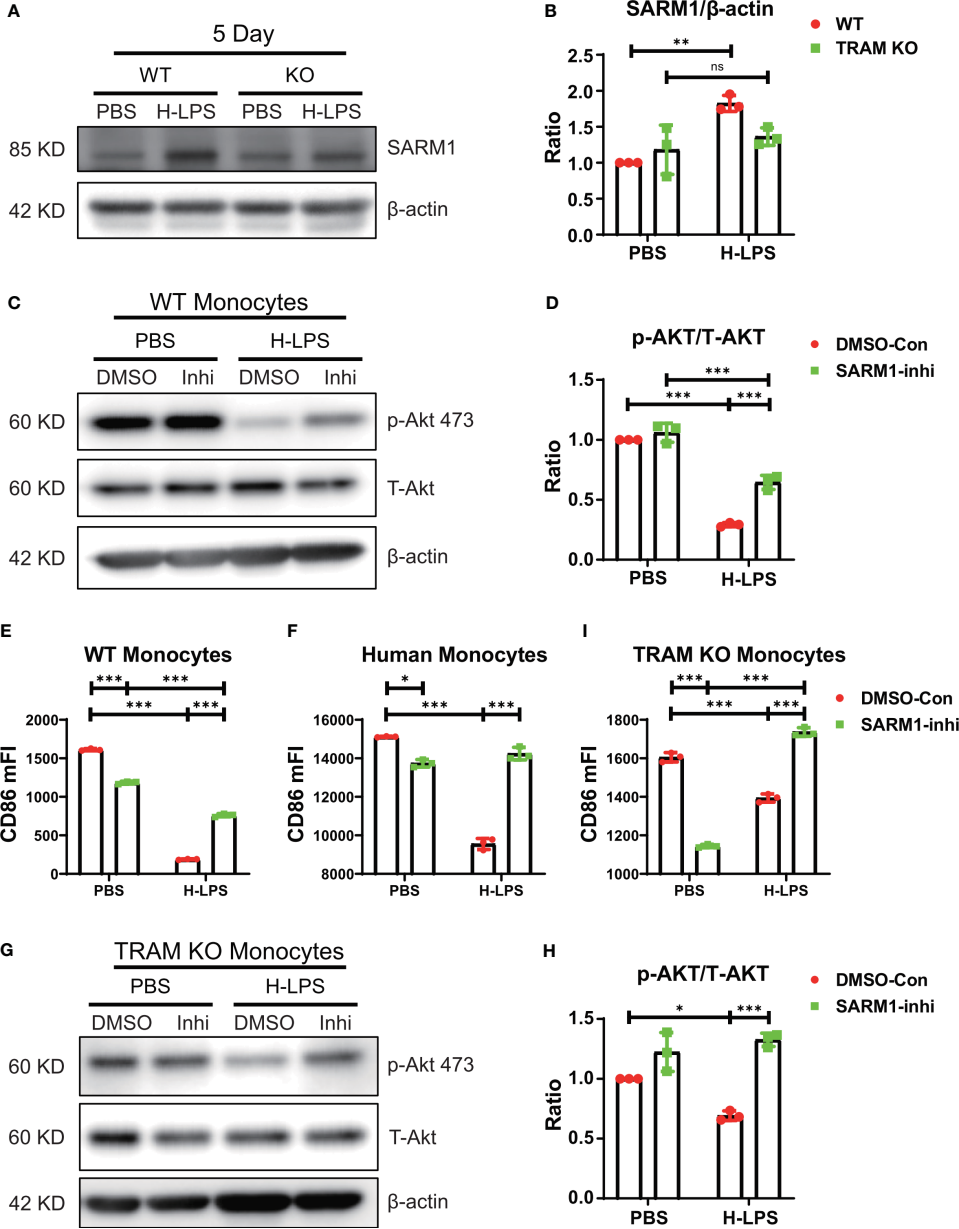
TRAM works synergistically with SARM1 in driving monocyte exhaustion. **(A, B)** BMDMs from WT and TRAM KO mice were stimulated with either PBS or H-LPS (100 ng/mL) for 5 days. The level of SARM1 expression was determined by western blot **(A)**. The relative level of SARM1 was normalized to β-actin **(B)**. **(C–E)** SARM1 inhibitor or DMSO control treated WT BMDMs were stimulated with either PBS or H-LPS (100 ng/mL) for 5 days. The levels of p-Akt and T-Akt expression was determined by western blot **(C)**. The relative level of p-Akt was normalized to T-Akt **(D)**. The expression of CD86 was analyzed with flow cytometry **(E)**. The CD86 expression of SARM1 inhibitor or DMSO control treated human monocytes **(F)** was analyzed with flow cytometry. Cells were stimulated with either PBS or H-LPS (100 ng/mL) for 5 days. **(G–I)** SARM1 inhibitor or DMSO control treated TRAM KO mice BMDMs were stimulated with either PBS or H-LPS (100 ng/mL) for 5 days. The levels of p-Akt and T-Akt expression was determined by western blot **(G)**. The relative level of p-Akt was normalized to T-Akt **(H)**. The expression of CD86 was analyzed with flow cytometry **(I)**. DMSO, DNSO Control; Inhi, SARM1 inhibitor. n=3. Error bars represent means ± SD. ns, not significant *p<0.05, **p<0.01, ***p<0.001.

As noted in **Figures 2**, **3**, TRAM deletion attenuated, but did not fully restore the activation of Akt/NFκB. Our data suggest that additional players may be collectively involved in the suppression of Akt/NFκB in exhausted monocytes. Among TLR4 intra-cellular adaptors, SARM1 is the only adaptor known to suppress NFκB activation (26). We performed western blot analyses and detected the presence of SARM1 in both wild type and TRAM deficient monocytes (**Figures 4A, B**). Prolonged LPS exhaustion treatment led to a further induction of SARM1 in wild type, but not TRAM deficient monocytes (**Figures 4A, B**). To test whether SARM1 may be collectively involved in the suppression of Akt/NFκB and CD86 expression, we applied a selective SARM1 inhibitor to the culture system. We observed that SARM1 inhibition led to a partial restoration of Akt/NFκB as well as the expression of CD86 in LPS-treated exhausted monocytes (**Figures 4C–E**; **Supplementary Figure S2**). SARM1 inhibitor also reduced the expansion of Ly6C^++^ population (**Supplementary Figures S3A, B**). Independent of the inhibitor approach, we applied either control or SARM1 specific siRNA to the culture system (**Supplementary Figure S3C**). We observed that the application of SARM1 specific siRNA reduced the cellular levels of SARM1 (**Supplementary Figure S3C**), and partially restored Akt activation in exhausted monocytes (**Supplementary Figures S3C, D**). We further validated our observation in human primary monocytes and observed that SARM1 inhibitor can partially restore the levels of CD86 in LPS treated monocytes collected from peripheral blood (**Figure 4F**). We then applied SARM1 inhibitor to TRAM deficient monocytes and observed that SARM1 inhibitor fully restored the levels of pAkt in TRAM deficient monocytes exhausted by LPS (**Figures 4G, H**). Similarly, we observed that SARM1 inhibitor fully restored the levels of CD86 in TRAM deficient monocytes exhausted by LPS (**Figure 4I**). SARM1 inhibitor also fully suppressed the expansion of Ly6C^++^ population as measured by flow cytometry (**Supplementary Figure S4**). Collectively, our data suggest that TRAM may work synergistically with SARM1 in establishing the state of monocyte exhaustion, and suppressing Akt/NFκB-mediated expression of immune-enhancing CD86 (**Supplementary Figure S4**).

### TRAM is involved in monocyte exhaustion *in vivo* during experimental sepsis

**Figure 5:**
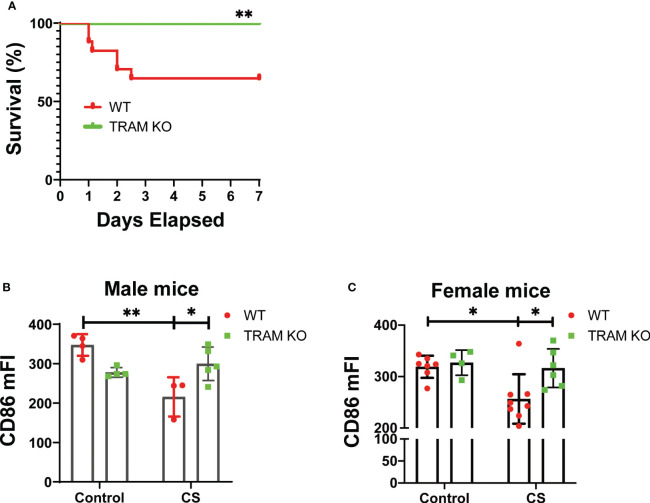
TRAM is involved in monocyte exhaustion *in vivo* during experimental sepsis. **(A)** The Kaplan-Meier survival plot of WT (17) and TRAM KO (16) male mice with sever sepsis induced by CS (~1.5 mg/g of BW) i.p. injection. *p<0.01. **(B, C)** WT and TRAM KO male **(B)** or female **(C)** mice were i.p. injected with CS (~0.9 mg/g of BW) to induce mild sepsis. The bone marrow monocytes CD86 expression were analyzed through flow cytometry at day 6 after CS injection. n=3-8 mice/group. Error bars represent means ± SD. *p<0.05, **p<0.01.

To test the pathological relevance of our mechanistic findings, we performed an experimental sepsis study with the well-adopted cecal slurry injection model (27, 28) which allows a precise titration of sepsis severity. We observed that a one-time injection of ~1.5 mg/g of CS led to significant mortality in wild type mice (**Figure 5A**). In contrast, TRAM deficient mice were protected and survived similar CS injection (**Figure 5A**). We then adopted a mild sepsis model with an injection of ~0.9 mg/g CS which allows 100% survival. 7 days post the initial CS injection, we harvested bone marrow monocytes and measured the levels of CD86 via flow cytometry. As shown in **Figures 5B, C**, CD86 levels were significantly reduced in wild type, but not TRAM deficient mice following the onset of mild sepsis. This phenotype was observed in both male and female mice (**Figures 5B, C**).

## Discussion

Monocytes play a crucial role in the immune response contributing to pathogen clearance and immune regulation. However, during septic insult, systemic and prolonged exposure to inflammatory stimuli such as LPS in circulation can lead to monocyte exhaustion, impairing their immune-enhancing functions and promoting immune suppression. Previous comparative analyses revealed key features of monocyte exhaustion, including reduced expression of immune-enhancing genes like CD86 and elevated expression of immune-suppressive genes such as PD-L1 (4, 5). We validated these key signatures with the well-established *in vitro* culture model with prolonged LPS challenges. We demonstrated that naïve monocytes treated with short-term LPS stimulation robustly express both CD86 and PD-L1 genes. However, prolonged LPS challenges result in the significant suppression of CD86 expression while sustaining PD-L1 expression.

Our studies further advance the emerging concept of monocyte exhaustion in contrast to the well-known concepts of the early phase of inflammation and the second phase of tolerance (4, 14, 29). Monocytes with a single challenge of high dose LPS are capable of robustly expressing a wide plethora of genes with pleiotropic functions. Following the initial phase of robust “cytokine storm” response, monocytes enter the second phase of “tolerance”, with dampened expression of acute inflammatory mediators (14). However, following the prolonged LPS challenges, monocytes not only retain certain features of tolerance with a sustained reduction of immune enhancing genes such as CD86, but also adopt additional exhaustion features characterized by sustained expression of selected pathogenic inflammatory mediators such as CD38 and S100A8 as well as immune suppressors such as PD-L1 (4). The first two phases of “cytokine storm” and “tolerance” have been well characterized, and were not the scope of our current study. In contrast, our current data reveal that exhaustion can only be developed by prolonged challenges with high dose LPS. The exhaustion phenotype induced by prolonged challenges with high dose LPS is also distinct from the low-grade immune-enhancing phenotype induced by prolonged challenges with subclinical low-dose LPS (9). Our current work was limited to the final phase of monocyte exhaustion selectively caused by prolonged challenges with high dose LPS. Given the complex and vast adaptation of innate monocytes dependent upon signal strength and duration, our current studies are limited in scope. Future systems analyses with detailed kinetics and time-courses are warranted in order to thoroughly clarify these important phenotypes as well as underlying mechanisms.

Complementing previous mechanistic studies regarding the critical role of TRAM in mediating cellular response to LPS (4, 30), we confirmed that TRAM is responsible not only for the initial induction of CD86 and PD-L1 but also for the sustained suppression of CD86 in exhausted monocytes following prolonged LPS challenges. Our functional assays validated that exhausted monocytes, generated through prolonged LPS challenge, exhibit potent immune-suppressive effects on T cell proliferation and activation. Notably, TRAM-deficient monocytes display limited T cell suppression, validating the role of TRAM in driving monocyte exhaustion and its functional consequences. Our data complements previous studies revealing that TRAM is an essential gate keeper for cellular responses to LPS, responsible for the wide arrays of signaling processes (9, 31) and broadly involved in all phases of monocyte activation ranging from the initial response to exhaustion (4, 9). Due to the focus of the current study, we did not address the “low-grade inflammatory and immune-enhancing” effects of monocytes trained by prolonged challenges with subclinical low dose LPS as examined in separate studies (9).

Our current study clarified the molecular mechanisms underlying sustained suppression of CD86 in exhausted monocytes. Previous studies reveal that Akt and NFκB are responsible for the induction of immune-enhancing CD86 (32, 33), and priming agents such as beta-glucan can potently elevate Akt and sustain the immune-enhancing state of primed monocytes (34). Our data reveal an important bi-phasic effect of LPS challenge, with an initial robust induction of Akt and NFκB during early LPS stimulation, coinciding with the induction of CD86 in naïve monocytes. However, as monocytes transition to a sustained exhaustion state, Akt and NFκB activation is drastically inhibited, corresponding to the suppressed expression of CD86. TRAM deletion attenuates this suppression, revealing TRAM’s role in mediating sustained Akt/NFκB suppression and the generation of immune-suppressive exhausted monocytes. To further uncover the molecular basis of Akt/NFκB suppression in exhausted monocytes, we characterized the sustained induction of TAX1BP1, a known suppressor of NFκB as well as the TRIF-pathway responsible for Akt activation (35–37). Through transcriptomic analysis and comparative analyses using independent scRNAseq datasets, we identified sustained elevation of TAX1BP1 expression in exhausted monocytes from both murine sepsis models and human sepsis patients. Our western blot analyses confirmed elevated protein levels of TAX1BP1 in exhausted monocytes, which are significantly attenuated in TRAM-deficient cells. Our data suggest that TRAM-mediated activation of Src and STAT1 enables sustained expression of TAX1BP1, leading to Akt/NFκB suppression and reduced expression of immune-enhancing genes like CD86.

Furthermore, our study uncovers a potential synergistic role between TRAM and SARM1, another TLR4 intracellular suppressor, in driving monocyte exhaustion. We validated the presence of SARM1 in monocytes and detected its further induction during prolonged LPS exhaustion. Our independent approaches using siRNA knockdown and selective SARM1 inhibitor collectively validated the role of SARM1 in partially contributing to the suppression of Akt/NFκB activation and CD86 expression in exhausted monocytes. This observation was validated in both murine and human monocytes. We observed that SARM1 inhibition can fully restore Akt activation and CD86 expression in Tram^-/-^ exhausted monocytes, revealing a synergistic role between TRAM and SARM1 in establishing monocyte exhaustion and suppressing immune-enhancing pathways.

Our *in vivo* studies with the cecal-slurry induced experimental sepsis model further provided pathological relevance of our mechanistic findings. We confirmed that TRAM-deficient mice are protected against severe morbidity/mortality associated with sepsis. Furthermore, we validated the reduction in CD86 levels in monocytes collected from wild-type mice but not TRAM-deficient mice following the onset of mild sepsis, highlighting TRAM’s involvement in monocyte exhaustion *in vivo*. However, we realize the limitation of our studies with the utilization of whole body TRAM knockout mice. We can not conclude conclusively the role of TRAM-mediated monocyte exhaustion as the sole cause of sepsis severity. Sepsis pathogenesis is believed to involve a wide array of immune cells and tissues with complex adaptation dynamics during distinct phases of sepsis pathogenesis. Intercellular communications among cells through extrinsic mechanisms may also be involved. Future studies with myeloid selective TRAM deletion will be needed to further clarify this issue.

In conclusion, our study provides some initial clues on the molecular mechanisms underlying sustained monocyte exhaustion *in vitro* and suggests the important role of TRAM adaptor signaling in this process. Our findings provide insights into the dynamic regulation of monocyte adaptation during the course of septic insult. Future studies with more refined animal models as well as human patient studies will be needed to further define the dynamics of sepsis pathogenesis in order to guide the development of therapeutic strategies targeting monocyte exhaustion in sepsis and other immune-related disorders.

## Materials and methods

### *In vitro* cell culture

Primary cells from either C57BL/6 (WT) or TRAM KO (Ticam2^-/-^) mice were used for *in vitro* cell culture in this study. C57BL/6 mice were originally from Jackson’s Laboratory and TRAM KO mice with C57BL/6 background were kindly provided by Dr. Holger Eltzschig (University of Texas Houston). All animal procedures were in accordance with the U.S. National Institutes of Health Guide for the Care and Use of Laboratory Animals and approved by Institutional Animal Care and Use Committee (IACUC) of Virginia Tech. Primary bone marrow cells were harvested from WT or TRAM KO mice as described before (8, 38). Primary bone marrow (BM) cells were cultured in complete RPMI 1640 media (10% PBS, 1% L-Glutamine, 1% penicillin-streptomycin and 10ng/mL M-CSF (PeproTech, Rocky Hill, #351-02)) at 37°C in a humidified 5% CO2 atmosphere. Cells were treated with high-dose LPS (H-LPS, 100ng/mL) or PBS control for 5 hours or 5 days during the culture. Fresh complete RPMI 1640 media with M-CSF and treatments were added every 2 days as reported before (8, 38). According to our previously characterized, bone marrow cells cultured under such conditions were loosely adherent, monocyte-like, and no mature macrophage marker CD71 expression (9).

### Human PBMC isolation and culture

Human blood was purchased from PBS Research Blood Components. Human PBMC were isolated using Ficoll-Paque Plus density gradient media (GE Healthcare Bio Science). In brief, The PBMC fraction was washed twice and resuspended in RPMI-1640, supplemented as described above, and the cell density was adjusted to 10^6^ cells/mL.

### T cell isolation and coculture

Mouse spleen CD3^+^ T cells were isolates by using MojoSort Mouse CD3 T Cell Isolation Kit (Biolegend, #480031) resulting in >95% purity. Isolated spleen CD3^+^ T cells were labeled with 5 μM carboxyfluorescein succinimidyl ester (CFSE; Invitrogen, #C34554) for 10 min at room temperature (RT) and then blocked with 3 mL FBS for 10 min at 37°C. CFSE-labeled T cells were resuspended in culture medium (RPMI 1640 supplemented with 10% PBS, 1% L-Glutamine, 1% penicillin-streptomycin and 10% PBS, 1% L-Glutamine, 1% penicillin-streptomycin and 0.055mM 2-mercaptoethanol) and stimulated with 2.5 μg/mL coated anti-CD3 antibody (Bio X Cell, #BE0001-1) and 2.5 μg/mL soluble anti-CD28 (Bio X Cell, #BE0015-1) antibody in U-bottom 96-well plates for 2 days. For coculture studies, CFSE-labeled purified T cells (1 × 10^6^ cells/mL) were mixed with 5-day PBS or H-LPS treated WT or TRAM KO monocytes at a ratio of 1:1 in the presence of coated anti-CD3 and soluble anti-CD28 antibody for 2 days in a U-bottom 96-well plates (200 μL/well).

### Murine sepsis model

Sepsis was induced in 6- to 8-week-old WT or TRAM KO mice by injecting cecal slurry intraperitoneally, as previously described (27, 28). Briefly, the ceca were obtained from 12-week-old C57BL/6 mice euthanized by cervical dislocation. The cecal contents were extracted, mixed with PBS (250 ug/µL), filtered through 860 µm and 190 µm mesh strainers in sequence, combined with an equal volume of 30% glycerol in PBS (final concentration 125 µg/µL), and stored at -80˚C. For the cecal slurry injections, the frozen cecal slurry (CS) stock was rapidly thawed in a 37˚C water bath and injected into the peritoneal cavity of the recipient mice at a dose of either ~1.5 mg/g (for severe sepsis mortality studies) or ~0.9 mg/g (for mild survival sepsis) of body weight for 7 days. At day-7, mice were euthanized by cervical dislocation and bone marrow femurs were isolated from mice for the future flow test.

### Western blot analysis

Western blot was performed as previously describes (39). Shortly, cultured cells were lysed by 2% lysis buffer with the cocktail of proteinase inhibitor and phosphatase inhibitor (Sigma, #P8340, #P5276, #P0044). Protein concentration of cell lysates were determined using the DC Protein Assay Kit (Bio-Rad, #5000112). Proteins were extracted by 10% Acrylamide gel and transferred to PVDF membranes. The electroblotted membranes were blocked by PBST buffer containing 5% non-fat milk (Bio-Rad, #170-6404) for 1 hours, followed by incubation with anti-STAT1 (Cell Signaling, #9172), anti-phospho-STAT1 (Cell Signaling, #9177), anti-Akt (Cell Signaling, #9272), anti-phospho-Akt 473 (Cell Signaling, #9271), anti-NFκB p65 (Cell Signaling, #8242), anti-phospho-NFκB p65 (Cell Signaling, #3031), anti-SARM1 (Invitrogen, #PA5-20059) anti-TAX1BP1 (Proteintech, #14424-1-AP), anti-Src (Cell Signaling, #2110), anti-phospho-Src (Cell Signaling, #2105), and anti-β-actin (Cell Signaling, #4970) primary antibody at 4°C overnight and secondary HRP-conjugated anti-rabbit IgG antibody (Cell Signaling, #7074) or HRP-conjugated anti-mouse IgG antibody (Cell Signaling, #7076) at room temperature for 1 hour. Images were developed with ECL detection kit (VWR, #490005-020), and the relative expressions of target protein were quantified with ImageJ (NIH).

### Flow cytometry

BM cells in single-cell suspension from *in vivo* experiments as well as murine monocytes, human monocytes and T cells from *in vitro* cultures were assessed with the flow cytometry. Cells were washed with PBS, harvested, and preincubated with FcBlock (anti-mouse CD16/32, BD Biosciences, #553141 or Human TruStain FcX, Biolegend, #422302). Cells were then stained with fluorochrome-conjugated antibodies against mouse CD11b (APC-Cy7, Biolegend, #101226), Ly6C (PE-Cy7, Biolegend, #128018), PD-L1 (APC, Biolegend, #124312), CD86 (FITC, Biolegend, #105006) or human CD66b (FITC, Biolegend, #305104), CD14(PE-Cy7, Biolegend, #301814), CD16 (APC-Cy7, Biolegend, #360710), CD86 (PE, Biolegend, #374206), PD-L1 (PE, Biolegend, #329706), for 30 minutes followed by washing with FACS buffer. Then cells were resuspended in FACS buffer containing Propidium Iodide (Invitrogen, #P3566). Samples were detected by FACS Canto II (BD Biosciences), and data were analyzed with FlowJo (Ashland, OR). SH800S cell sorter (SONY) was used for monocytes sorting.

### scRNA-seq data re-analysis

Publically available processed scRNAseq data sets, representing monocyte exhaustion *in vitro* reported previously by our group (4); human sepsis scRNAseq data set reported by Reyes et al. (25) and murine cecal ligation and puncture sepsis dataset reported by Zhang et al. (22) were re-analyzed as we described (4, 9). Briefly, relative expression levels of selected targets were evaluated with the Seurat software under the unbiased default setting, representing the percentages of target gene expression among the cell sub-populations as well as normalized expression levels of the target gene as we previously described (4, 9). Key target genes differentially expressed within distinct clusters were analyzed using the non-parametric Wilcoxon rank sum test in R. The dot size represents the percentage of cell population expressing the target gene and the dot color intensity represents the normalized expression level of the given target.

### Statistical analysis

GraphPad Prism v8.0 was used to generate graphs and perform statistical analysis. Student’s *t*-test (two groups) or two-way ANOVA analysis (multiple groups) were used to determine the significance. Statistically significant results (*p*) are indicated as: *p<0.05; **p<0.01; ***p<0.001.

## Data availability statement

The datasets presented in this study can be found in online repositories. The names of the repository/repositories and accession number(s) can be found in the article/**Supplementary Material**.

## Ethics statement

Ethical approval was not required for the studies involving humans because The human blood cells are purchased from a commerical source without any personal identifier. Therefore, they are not qualified as human subject research. The studies were conducted in accordance with the local legislation and institutional requirements. The human samples used in this study were acquired from a by- product of routine care or industry. Written informed consent to participate in this study was not required from the participants or the participants’ legal guardians/next of kin in accordance with the national legislation and the institutional requirements. The animal study was approved by Institutional Animal Care and Use Committee (IACUC) of Virginia Tech. The study was conducted in accordance with the local legislation and institutional requirements.

## Author contributions

JW: Data curation, Funding acquisition, Writing – original draft, Writing – review & editing. YW: Data curation, Writing – review & editing. RL: Data curation, Writing – review & editing. YZ: Data curation, Writing – review & editing. LL: Data curation, Funding acquisition, Investigation, Methodology, Supervision, Writing – original draft, Writing – review & editing.
