# Supplementary material for: TRAM deletion attenuates monocyte exhaustion and alleviates sepsis severity
Source: Front Immunol. 2023 Dec 15;14:1297329. doi: 10.3389/fimmu.2023.1297329 (PMC10756061; doi:10.3389/fimmu.2023.1297329)
Supplement: Supplementary file 1 [file DataSheet_1.pdf]

Supplementary information

TRAM deletion attenuates monocyte exhaustion and alleviates sepsis severity

Jing Wang[1], Yajun Wu, RuiCi Lin, Yao Zhang, Liwu Li[1]*

[1]Department of Biological Sciences, Virginia Tech, Blacksburg, VA24061

Keywords: Monocyte exhaustion, TRAM, sepsis, intervention, signaling

Running Title: TRAM-mediated monocyte exhaustion during sepsis

*Correspondence:

Liwu Li

970 Washington Street

Virginia Tech

Blacksburg, VA 24061-0910

Email: lwli@vt.edu

Figure S1

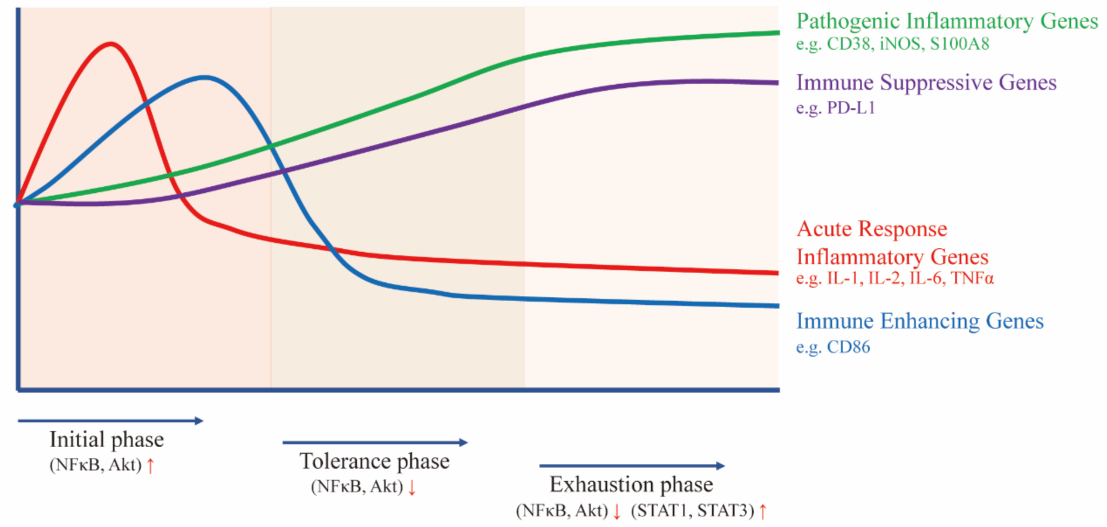

Figure S1 Illustration of distinct phase of monocyte adaptation ranging from an initial inflammation followed by tolerance and eventual exhaustion.

Figure S2

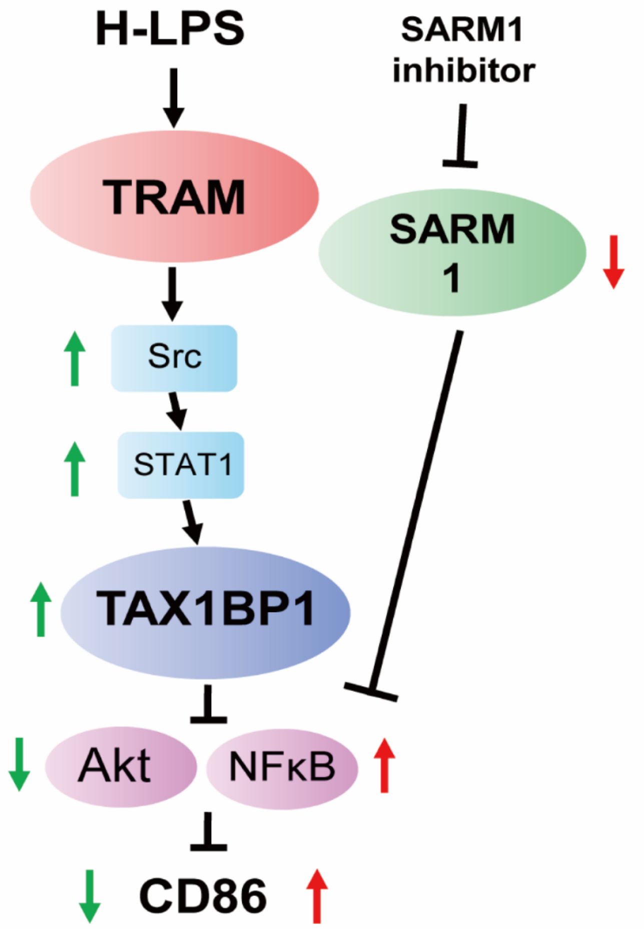

Figure S2 TRAM works synergistically with SARM1 in establishing the state of monocyte exhaustion. TRAM works with SARM-mediated activation of Src/STAT1 enables sustained expression of TAX1BP1, leading to the suppression of Akt/NFκB p65 and the reduced expression of immune-enhancing genes CD86. SARM1 inhibition led to a partial restoration of Akt/NFκB p65 as well as the expression of CD86 in LPS-treated exhausted monocytes. SARM1 inhibitor synergistically with TRAM deficient fully restored the levels of CD86 in exhausted monocytes.

Figure S3

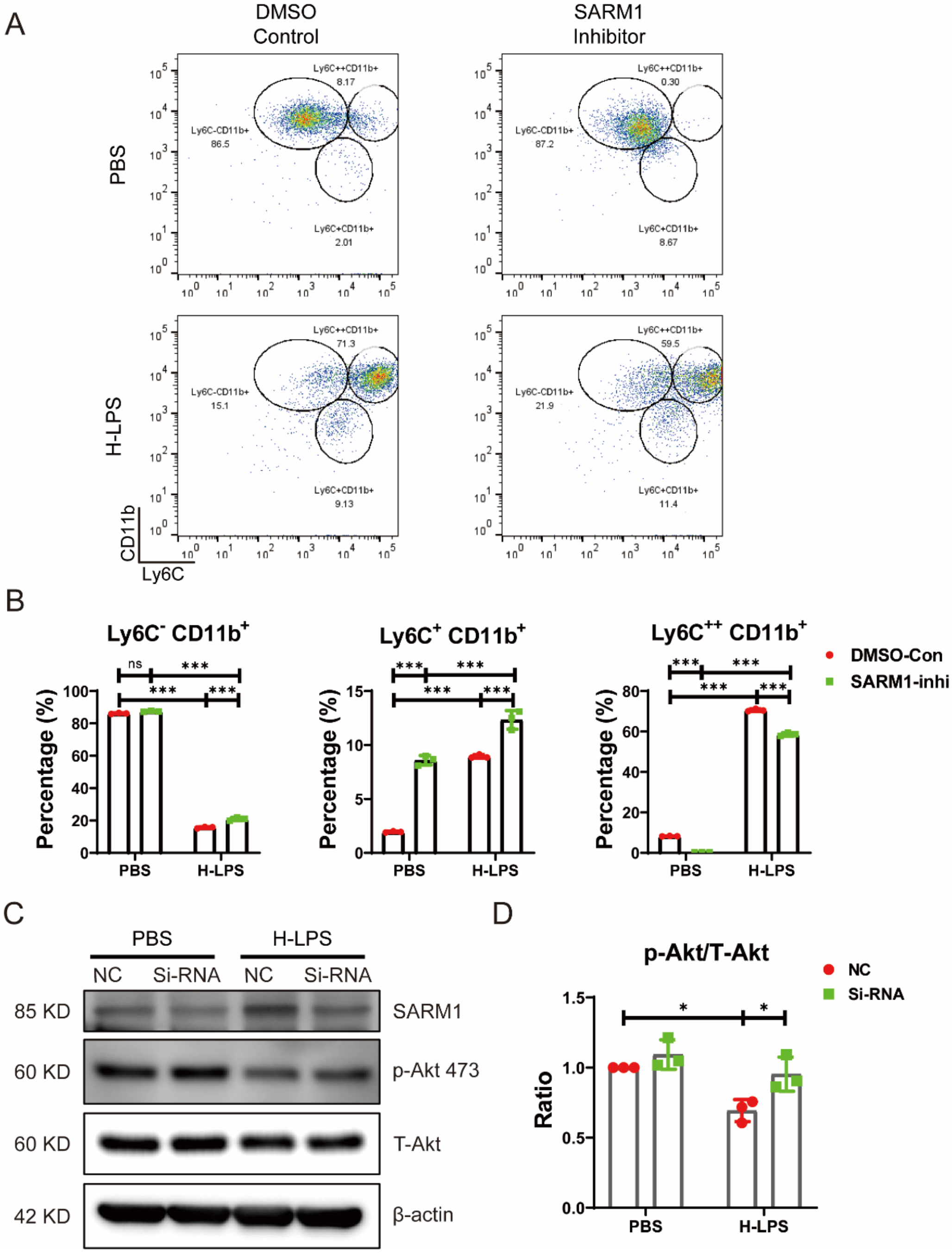

Figure S3 SARM1 knock-down partially restores Akt. SARM1 inhibitor or DMSO control treated WT BMDMs were stimulated with either PBS or H-LPS (100 ng/mL) for 5 days. (A) $Ly6C^- CD11b^+$, $Ly6C^+ CD11b^+$, $Ly6C^{++} CD11b^+$ populations were analyzed with flow cytometry. (B) The frequencies of indicated populations of WT BMDMs were quantified. The flow cytometry data are representative of at least three independent experiments. (C) SARM1 siRNA pre-treated WT BMDMs were stimulated with either PBS or H-LPS (100 ng/mL) for 5 days. The expression levels of SARM1, p-Akt and T-Akt was determined by western blot. (D) The relative level of p-Akt were normalized to T-Akt. n=3. Error bars represent means ± SD. *p<0.05, **p<0.01, ***p<0.001.

Figure S4

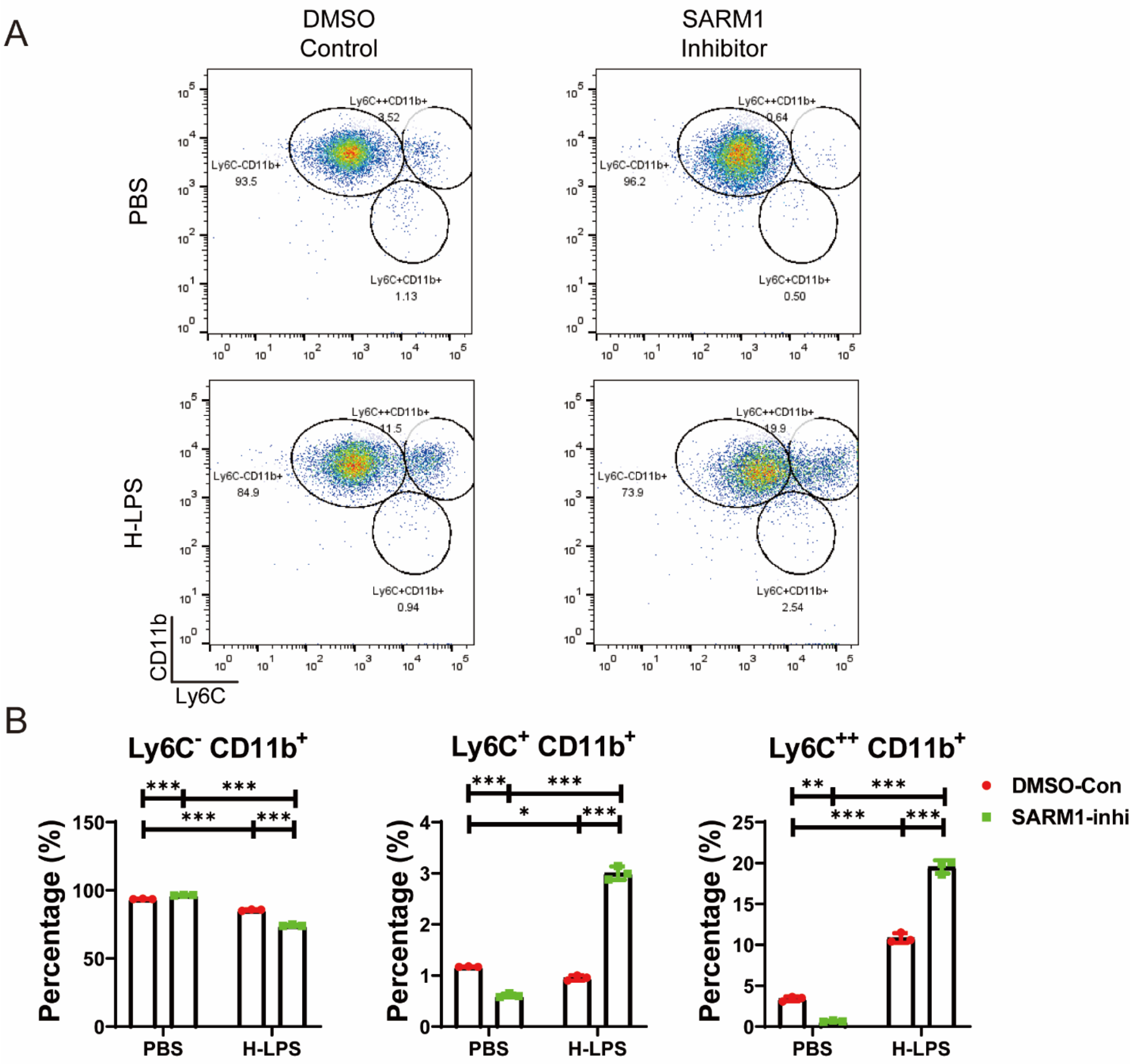

Figure S4 SARM1 inhibitor together with TRAM KO completely restores Akt and CD86. SARM1 inhibitor or DMSO control treated TRAM KO mice BMDMs were stimulated with either PBS or H-LPS (100 ng/mL) for 5 days. (A) Ly6C$^-$ CD11b$^+$, Ly6C$^+$ CD11b$^+$, Ly6C^{++} CD11b$^+$ populations were analyzed with flow cytometry. (B) The frequencies of indicated populations of WT BMDMs were quantified. The flow cytometry data are representative of at least three independent experiments. n=3, and error bars represent means ± SD. *$p<0.05$, **$p<0.01$, ***$p<0.001$.
